# Phrenic Paralysis with Transposition of the Heart

**Published:** 1898-09

**Authors:** Charles G. Stockton

**Affiliations:** Buffalo, N. Y.


### PHRENIC PARALYSIS WITH TRANSPOSITION OF THE HEART.—CLINICAL REPORT WITH PRESENTATION OF CASE.[1]

By CHARLES G. STOCKTON, M. D., Buffalo, N. Y.

THIS man came into the wards of the Buffalo General Hospital about four weeks ago apparently suffering from grippe. He had moderate cough, slight temperature and deficiency in vesicular breathing sounds and some broncho-vesicular breathing. The man's heart was very much depressed, both as to frequency and force. In fact when he sat up the pulse was hardly perceptible and when lying down the heart sounds could not be heard in the ordinary area. After a careful examination I found that this depended partly upon displacement of the heart, which was transposed to the right side. The apex could not be felt at all for two or three weeks, but the first sound could be heard, with greatest distinctness at the apex, just at the left of the border of the sternum; and about the third interspace, to the right of the sternum, could be heard the semilunar second sounds. Percussion confirmed this view that the heart was moved to the right.

It seemed a little difficult to account for this transposition of the heart, but after a time it was noticed that the diaphragm was also displaced, or had receded upward and was motionless in respiration. If anything, the diaphragm was elevated during the respiratory act rather than depressed. The stomach was found to be entirely above the free border of the ribs. The liver was also elevated considerably, and I was forced to account for this peculiar loss of power of the diaphragm and this malposition of the thoracic viscera. The case presented some perplexity at first, as I was not prepared to believe that the man had phrenic paralysis, because he had so little dyspnea, but I have changed my opinion and come to the conclusion that the man has phrenic paralysis; that he had a neuritis of the phrenic nerve and that has been, I think, proven by subsequent events.

I think that you can notice the peculiarities of the abdomen and thorax that I have mentioned. The fact that led me to believe that this man had paralysis of the phrenic nerve was, first, the lack of power in the diaphragm, which, after a time, as the man improved in health, was somewhat restored and is gradually improving. The second point was that he developed a neuritis of the right leg and still has a certain loss of power, a certain amount of toe drop and diminu-

---

1. Presented at the thirtieth annual meeting of the Medical Association of Central New York, at Buffalo, October 19, 1897.

tion both in sensation and motion of the right foot. The one thing that still troubles me is to account for the transposed heart. As you will perceive, it has now returned nearly into the normal position, as the diaphragm has begun to resume its function. I think the stomach is now nearly in its position. At first the stomach was at the upper border of the third rib, and the heart at the apex was to be heard at the left border of the sternum at about the third interspace, and the heart sounds were, for the most part, heard to the right of the sternum.

I think we must admit that some other process is in operation here aside from the loss of the use of the diaphragm. I can understand how the viscera might be advanced upward from this process. I cannot understand how the heart could be transposed to the right. It so happened that this man had an injury from a trolley car some years ago from which he lost his elbow joint, and at the same time he had his right ribs fractured, and I have presumed that following that he had an adhesive pleurisy, which possibly still exerts a certain amount of traction so displayed that when the loss of use of the diaphragm came about, this traction power pulled to the right when the diaphragm was advanced upward.

I have had the opportunity of seeing three cases of transposition of the heart to the right side, depending upon traction from adhesive pleurisy, and two of these cases came to post mortem, so I know that such cases exist, as, of course, is generally conceded. I must admit that the heart is assuming its natural position and the stomach is very much in position today.

